# Simple Assessment of Nitrogen Nutrition Index in Summer Maize by Using Chlorophyll Meter Readings

**DOI:** 10.3389/fpls.2018.00011

**Published:** 2018-01-19

**Authors:** Ben Zhao, Syed Tahir Ata-Ul-Karim, Zhandong Liu, Jiyang Zhang, Junfu Xiao, Zugui Liu, Anzhen Qin, Dongfeng Ning, Qiuxia Yang, Yonghui Zhang, Aiwang Duan

**Affiliations:** ^1^Key Laboratory of Crop Water Use and Regulation, Ministry of Agriculture, Farmland Irrigation Research Institute, Chinese Academy of Agricultural Sciences, Xinxiang, China; ^2^Key Laboratory of Soil Environment and Pollution Remediation, Institute of Soil Science, Chinese Academy of Sciences, Nanjing, China; ^3^Computer Engineering School, Weifang University, Weifang, China

**Keywords:** nitrogen nutrition index, summer maize, nitrogen diagnosis, chlorophyll meter, positional differences chlorophyll meter index

## Abstract

Rapid and non-destructive diagnostic tools to accurately assess crop nitrogen nutrition index (NNI) are imperative for improving crop nitrogen (N) diagnosis and sustaining crop production. This study was aimed to develop the relationships among NNI, leaf N gradient, chlorophyll meter (CM) readings gradient, and positional differences chlorophyll meter index [PDCMI, the ratio of CM readings between different leaf layers (LLs) of crop canopy] and to validate the accuracy and stability of these relationships across the different LLs, years, sites, and cultivars. Six multi-N rates (0–320 kg ha^−1^) field experiments were conducted with four summer maize cultivars (Zhengdan958, Denghai605, Xundan20, and Denghai661) at two different sites located in China. Six summer maize plants per plot were harvested at each sampling stage to assess NNI, leaf N concentration and CM readings of different LLs during the vegetative growth period. The results showed that the leaf N gradient, CM readings gradient and PDCMI of different LLs decreased, while the NNI values increased with increasing N supply. The leaf N gradient and CM readings gradient increased gradually from top to bottom of the canopy and CM readings of the bottom LL were more sensitive to changes in plant N concentration. The significantly positive relationship between NNI and CM readings of different LLs (*LL*1 to *LL*3) was observed, yet these relationships varied across the years. In contrast, the relationships between NNI and PDCMI of different LLs (*LL*1 to *LL*3) were significantly negative. The strongest relationship between PDCMI and NNI which was stable across the cultivars and years was observed for PDCMI1−3 (NNI = −5.74 × PDCMI1−3+1.5, *R*^2^ = 0.76^**^). Additionally, the models developed in this study were validated with the data acquired from two independent experiments to assess their accuracy of prediction. The root mean square error value of 0.1 indicated that the most accurate and robust relationship was observed between PDCMI1–3 and NNI. The projected results would help to develop a simple, non-destructive and reliable approach to accurately assess the crop N status for precisely managing N application during the growth period of summer maize crop.

## Introduction

Nitrogen nutrition index (NNI) is the most widely recognized diagnostic tool used for accurately diagnosing the in-season crop N status (Lemaire et al., 2008). Recent studies showed that NNI has potential for in-season estimation of grain yield, grain amylose and protein contents, crop N requirement, photosynthesis capacity, crop N partition, and N use efficiency (Hu et al., 2014; Zhao, 2014; Ata-Ul-Karim et al., 2016a, 2017a,b; Dordas, 2017). However, due to the destructive and time-consuming plant sampling prerequisite for assessment of NNI, its application on farm level is restricted (Zhao et al., 2016). Attempts have been made to integrate the NNI with chlorophyll meter (CM) readings and canopy spectral measurements which can deliver scientists with non-destructive, timely and valuable information for application of NNI on large scale for in-season prediction crop N status, crop grain yield and quality (Mistele and Schmidhalter, 2008; Ata-Ul-Karim et al., 2017c).

Chlorophyll meter is an effective tool to assess crop N status (Yuan et al., 2016; Zhao et al., 2016). CM readings can be used to measure the relative greenness of plants and to positively correlate leaf chlorophyll with leaf N concentration in maize (Ziadi et al., 2008). However, several factors such as plant growth stage, cultivar, specific leaf weight, leaf thickness, leaf position on the plant, measurement location on a leaf as well as environmental stresses and solar radiation could significantly affect CM readings (Ziadi et al., 2008; Ata-Ul-Karim et al., 2016b; Zhao et al., 2016). Variations in these factors lead to the relatively poor relationships between CM readings and leaf N concentration or leaf chlorophyll (Bullock and Anderson, 1998). Several studies have attempted to use the adjusted CM readings (the ratio between CM readings and specific leaf weight) to overcome the variations associated with aforementioned factors while establishing the relationships between CM readings and leaf N concentration or leaf chlorophyll. Although the adjusted CM readings can significantly improve the estimation of leaf N concentration, yet this estimation is complex, time-consuming, and destructive as compared to the unadjusted CM readings (Peng et al., 1993).

Owing to the limitations of the NNI and CM readings for their application in crop N diagnosis, an alternative approach was developed to establish the relationship between CM or relative CM and NNI to evaluate crop N status (Debaeke et al., 2006; Ziadi et al., 2008). The relative CM value was obtained by dividing the CM readings from the test area by the readings from a saturated plot that has received a high N rate. The relationships between CM readings and NNI have been established in rice, wheat, maize, and barley, but these relationships change with location, year, and cultivar (Prost and Jeuffroy, 2007; Ziadi et al., 2008; Yuan et al., 2016; Zhao et al., 2016). Debaeke et al. (2006) reported that the relationship between relative CM readings and the NNI was not significantly affected by growth stage, year, or cultivar in wheat; however, Ziadi et al. (2008) reported that the response curves varied among sites and years in spring maize when relative CM readings were related to the NNI. Furthermore, the prerequisite of a high N treatment rate as a control also limits the application of relative CM readings for managing crop N status.

An alternative approach to estimate NNI is to use the difference of N concentration between the upper and lower leaf layers (LLs) of crop canopy (Zhao et al., 2016). Previous studies reported that the vertical N gradient of the canopy exists in several crops due to the non-uniform distribution of irradiance within canopies and due to a gradient of leaf ages in the canopy profile (Lemaire and Gastal, 2009). Dreccer et al. (2000) reported that during crop growth period, the gradient of N vertical distribution was more constant under the high N condition than low N condition. The previous study on rice indicated the large differences in the sensitivity of the response to increased N rates between the upper and lower LLs (Wang et al., 2006). Attempts have been made to establish the relationships between the NNI and CM or relative CM readings on a single leaf basis in various crops, yet the results were inconsistent (Debaeke et al., 2006; Ziadi et al., 2008). Zhao et al. (2016) reported that the relationship between NNI and the positional difference chlorophyll meter index (PDCMI; ratio of CM readings between different LLs of crop canopy) was not significantly affected by site, year, cultivar, and growth stage in winter barley. This approach of relating the ratio of CM readings to the NNI has not yet been tested in summer maize. Therefore, this study was endeavored to develop the relationships among NNI, leaf N gradient, CM readings gradient, and PDCMI across different LLs, and to validate the accuracy and stability of these relationships under different years, sites, and cultivars. The projected results would help to develop a simple, non-destructive and reliable approach to accurately assess the crop N status for precisely managing N application during the growth period of summer maize crop.

## Materials and methods

### Experimental design

Six multi-N application rate (0–320 kg ha^−1^) field experiments with four summer maize cultivars (Zhengdan958, Denghai605, Xundan20, and Denghai661) were conducted at two different experimental sites (Xinxiang, 35°18′N, and 113°52′E; Qinyang, 35° 08′N, and 112° 92′E) located in China (Table 1). The weather conditions of both experimental sites during 2015 and 2016 are shown in Table 2. The soil samples of top 0–20 cm soil layer were collected before planting. The air-dried and sieved soils samples were used to measure total N (traditional Kjeldahl method; Bremner and Mulvancy, 1982), Olsen-P (0.05 mol L^−1^ NaHCO_3_; Olsen et al., 1954), NH_4_OAc-K^+^ (1 mol L^−1^ ammonium acetate at pH 7; van Reeuwijk, 1992), and organic matter (Walkley–Black titration method; Nelson and Sommers, 1982). In each experiment, the treatments were arranged in a randomized complete block design with three replications. Fifty percent of the total N fertilizer (Urea) was applied before sowing while the remaining 50% was applied at the V6 stage (V6 stage indicates the sixth leaf of more than 50% plant fully expanded in the field, Table 1). All the plots received adequate quantities of triple superphosphate (150 kg P_2_O_5_ ha^−1^) and potassium chloride (120 kg K_2_O ha^−1^) before sowing. The plot size in all the experiments was 60 m^2^. The maize was over-seeded with hand planter and then thinned to a stand of 75,000 plants ha^−1^ and 60,000 plants ha^−1^ during 2015 and 2016, respectively, at the seedling stage. The field after sowing of summer maize was irrigated with a 60 mm irrigation water to ensure the emergence of summer maize. Due to the adequate rainfall during the growth period, the summer maize crop does not require further irrigation to fulfill crop water requirement. Chemical method was used to control weeds, pests, and diseases. The major limiting factor was the amount of N fertilizer applied.

**Table 1:** Basic information about the six field experiments conducted during 2015 and 2016 growth years at Xinxiang and Qinyang.

**Experiment No**.	**Sowing/Harvesting date**	**Soil properties**					**Cultivar**	***N*** **(kg N ha^−1^)**	**Sampling stage**
		**Soil type**	**Soil organic matter**	**Soil total N**	**Soil olsen-P**	**Soil NH4oAc-K^+^**			
Experiment 1	8-Jun	Light loam soil	12.26 g kg^−1^	0.74 g kg^−1^	35.67 mg kg^−1^	84 mg kg^−1^	Zhengdan958	0 (N0)	V6
2015	25-Sept						(ZD958)	75 (N1)	V9
Xinxiang								150 (N2)	V12
								225 (N3)	
								300 (N4)	
Experiment 2	8-Jun	Sandy light loam soil	10.43 g kg^−1^	0.61 g kg^−1^	33.94 mg kg^−1^	76 mg kg^−1^	Denghai605	0 (N0)	V6
2015	25-Sept						(DH605)	75 (N1)	V9
Xinxiang								150 (N2)	V12
								225 (N3)	
								300 (N4)	
Experiment 3	6-Jun	Light loam soil	14.2 g kg^−1^	0.83 g kg^−1^	44 mg kg^−1^	90 mg kg^−1^	Zhengdan958	0 (N0)	V6
2016	22-Sep						(ZD958)	90 (N1)	V9
Xinxiang								180 (N2)	V12
								270 (N3)	
Experiment 4	6-Jun	Light loam soil	9.5 g kg^−1^	0.57 g kg^−1^	23.51 mg kg^−1^	58.45 mg kg^−1^	Denghai605	0 (N0)	V6
2016	22-Sept						(DH605)	90 (N1)	V9
Xinxiang								180 (N2)	V12
								270 (N3)	
Experiment 5	15-Jun	Sandy soil	8.8 g kg^−1^	0.53 g kg^−1^	11.1 mg kg^−1^	62.8 mg kg^−1^	Xundan20	0 (N0)	V6
2015	24-Sept						(XD20)	80 (N1)	V9
Qinyang								160 (N2)	V12
								240 (N3)	
								320 (N4)	
Experiment 6	9-Jun	Sandy soil	9.3 g kg^−1^	0.56 g kg^−1^	12.5 mg kg^−1^	67.4 mg kg^−1^	Denghai661	0 (N0)	V6
2016	26-Sept						(DH661)	75 (N1)	V9
Qinyang								150 (N2)	V12
								225 (N3)	
								300 (N4)	
				

*V6, V9, and V12 stages indicate the sixth, ninth and twelfth leaf fully expanded of more than 50% plants in the field, respectively*.

**Table 2:** Total monthly precipitation, total monthly sunshine and mean monthly temperature during the 2015 and 2016 growth years of summer maize.

**Year**	**Site**	**Month**	**Total monthly precipitation (mm)**	**Total monthly sunshine (h)**	**Mean monthly temperature (°C)**
2015	Xinxiang	June	38	195	24.5
		July	85	255	26.2
		August	101	193	27.3
		September	119	175	20
	Qinyang	June	96	175	25.8
		July	64	238	26.9
		August	81	194	27.5
		September	74	187	22.7
2016	Xinxiang	June	95	219	22.7
		July	105	207	30.1
		August	71	214	28.7
		September	90	183	23.8
	Qinyang	June	84	176	21.8
		July	95	207	26.2
		August	82	194	27.1
		September	69	188	22.3

### Plant sampling and N determination

**Figure 1:**
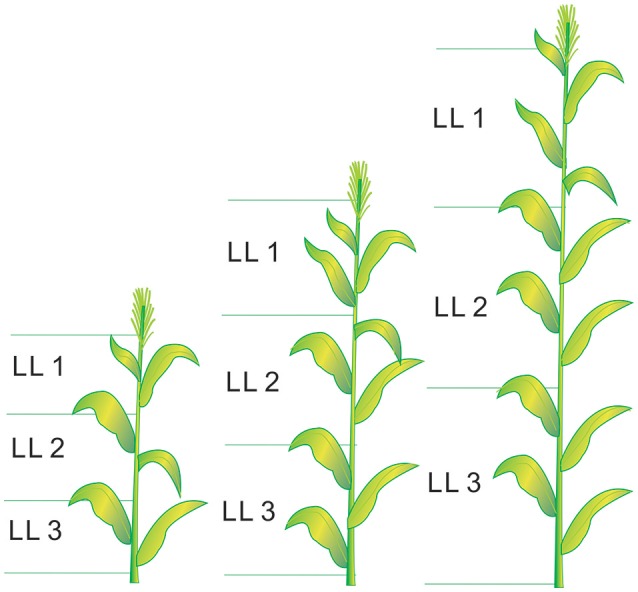
The schematic diagram of leaf layers (LLs) distribution from V6 to V12 growth stages in summer maize.V6 and V12 represent the sixth leaf and twelfth leaf, respectively.

Six plants from each plot were destructively sampled at different growth stages (from V6 to V12, V12 stage indicates the twelfth leaf of more than 50% plant fully expanded in the field) for growth analysis, following the approach of Mansouri-Far et al. (2010). The sampling stages for each experiment are presented in Table 1. The plant leaves were divided into three LLs (LL1, LL2, and LL3 from the top). The leaf number of each LL was calculated as the average value of all the fully expanded leaves at each sampling stage. The leaf number of each LL were 2, 3, and 4 at the V6, V9, and V12 growth stages, respectively (V9 stage indicates the ninth leaf of more than 50% plant fully expanded in the field). The schematic diagram of LL distribution is shown in Figure 1. The plant samples were heated for 30 min at 105°C to halt metabolism and then dried at 70°C to constant weight. Plant aboveground biomass was determined by summing the aboveground biomass of leaves (LL1, LL2, and LL3) and stem. The samples were ground before passing them through a 1 mm sieve in a Wiley mill. The samples were then stored in plastic bags at room temperature for chemical analysis. A subsample was taken from the ground samples to determine the N concentrations of different LLs and stem by a traditional Kjeldahl method (Bremner and Mulvancy, 1982). Leaf N concentration (LNC) was calculated as leaf N accumulation (LNA) divided by leaf biomass as follows:

(1) $$\text{LNA}=\frac{\text{LL}1_{\text{N}}\text{LL}1_{\text{B}}+\text{LL}2_{\text{N}}\text{LL}2_{\text{B}}+\text{LL}3_{\text{N}}\text{LL}3_{\text{B}}}{100}$$

(2) $$\text{LNC}=\frac{\text{LNA}}{\text{LL}1_{\text{B}}+\text{LL}2_{\text{B}}+\text{LL}3_{\text{B}}}$$

where LL_B_ is biomass (t ha^−1^) from LL1 to LL3, and LL_N_ is the N concentration of the corresponding LL.

Leaf N gradient was calculated as the difference in leaf N concentration between different LLs (LL1 to LL3) as follows:

(3) LL1−2=LL1−LL2

(4) LL1−3=LL1−LL3

(5) LL2−3=LL2−LL3

where LL1–2, LL1–3, and LL2–3 are the difference in leaf N concentration between LLs (LL1 to LL3) of the canopy, respectively (Wang et al., 2005).

Plant N concentration was calculated as plant N accumulation (PNA) divided by plant aboveground biomass as follows:

(6) $$\text{PNA}=\text{LNA}+{\text{S}}_{\text{N}}{\text{S}}_{\text{B}}$$

(7) $$\text{PNC}=\frac{\text{LNA}+{\text{S}}_{\text{N}}{\text{S}}_{\text{B}}}{\text{LL}1_{\text{B}}+\text{LL}2_{\text{B}}+\text{LL}3_{\text{B}}+{\text{S}}_{\text{B}}}$$

where *S*_*B*_ is stem biomass (t ha^−1^), and *S*_*N*_ is the stem N concentration.

### SPAD measurements

Chlorophyll meter (CM) readings at each sampling stage were recorded with a SPAD-502 (Minolta Camera Co., Osaka, Japan). CM readings were measured from six randomly selected plants from each plot. Three CM readings per leaf (one value around the midpoint of the leaf blade and two values 3 cm apart from the midpoint) were averaged as the mean CM reading for the leaf (Peng et al., 1993). The CM readings of each LL were calculated as the mean value of the CM readings of all the leaves in the LLs, respectively. CM readings of LL1 to LL3 from the top to bottom of the canopy were named CM1, CM2, and CM3, respectively. The difference of CM readings between different LLs (LL1 to LL3) was defined as CM1–2, CM1–3, and CM2–3, respectively. The PDCMI of different LLs was calculated by using the following equation:

(8) $${\text{PDCMI}}_{\text{ij}}=\frac{{\text{CM}}_{\text{i}}-{\text{CM}}_{\text{j}}}{{\text{CM}}_{\text{i}}+{\text{CM}}_{\text{j}}}$$

where CM_*i*_ and CM_*j*_ are the CM readings from LL1 to LL3, and i and j represent the LL number (1–3) and i < j.

### Nitrogen nutrition index

The N_c_ concentration of summer maize was described by using Equation (9), which is the most widely accepted N_c_ curve of maize (Plénet and Lemaire, 2000). The NNI was calculated as the ratio of plant N concentration and N_c_ based on the same plant aboveground biomass (AGB) of summer maize as follows:

(9) $${\text{N}}_{\text{c}}=3.4{\left(\text{AGB}\right)}^{-0.37}$$

(10) $$\text{NNI}=\frac{\text{PNC}}{{\text{N}}_{\text{c}}}$$

When NNI = 1, N nutrition was considered optimal, while NNI > 1 and NNI < 1 indicated excess and deficient N nutrition, respectively.

### Statistical analysis

The data obtained in the present study were analyzed by the univariate ANOVA method using the SPSS-13 software (SPSS Inc., Chicago, IL, USA). The difference between treatments means was assessed by using least significant difference test at 95% level of significance. The year, cultivar, growth period, and N treatments were treated as fixed factors.

A linear regression analysis was performed by the least squares regression method using SPSS-13 software to establish the relationships among leaf N gradient, CM readings, PDCMI, and NNI. The NNI was dependent variable while leaf N gradient, CM readings, and PDCMI were independent variables. A simple linear regression with groups was performed using GenStat-12 software (VSN International Ltd., Cambridge, England). The coefficient of determination (*R*^2^) can explain how much of the variability of independent variable can be caused by its relationship to the dependent variable. The higher the *R*^2^-value, the better the fit.

### Model development and validation

The data sets acquired from experiments 1 to 4 (Xinxiang) were used to develop the relationships while the data sets acquired from of experiments 5 and 6 (Qinyang) were used to validate these relationships (Table 1, the data was shown as Supplementary Material). As the individual prediction models are generally significant for a specific location, cultivar and year, but might have poor predictive performance under different locations and cultivars. Therefore, the selection of data for development (experiment 1–4) and validation (experiment 5–6) of the models was intended to ensure the applicability prediction models developed in the present study for different locations and cultivars. The root mean square error (RMSE) between predicted and observed values were used to test the goodness of fit of the linear regression relationships between CM readings, PDCMI and NNI. The RMSE was calculated as follows:

(11) $$\text{RMSE}=\sqrt{\frac{\sum_{1}^{\text{n}}{\left({\text{P}}_{\text{i}}-{\text{O}}_{\text{i}}\right)}^{2}}{\text{n}}}$$

where n is the number of samples, *P*_*i*_ is the estimated value of the relationship, and *O*_*i*_ is the observed value from the validation data. The lower the RMSE value, the better the validation.

A simple linear regression with groups was used to determine whether the NNI- CM readings and NNI-PDCMI relationships were stable across the years and cultivars according to the methodology proposed by Mead et al. (1993). Similar methodology using the linear curve with the following model has also been previously reported by Ziadi et al. (2008) and Zhao et al. (2016):

(12) Y=aX+b

where Y and X are the response variable and explanatory variable, respectively. The parameters a and b are the slope and intercept of the linear curve, respectively. Cultivar and year were the factors of the groups analyzed in this study.

## Results

### Dynamic changes in the NNI with year, cultivar, growth stage, and N rate

**Figure 2:**
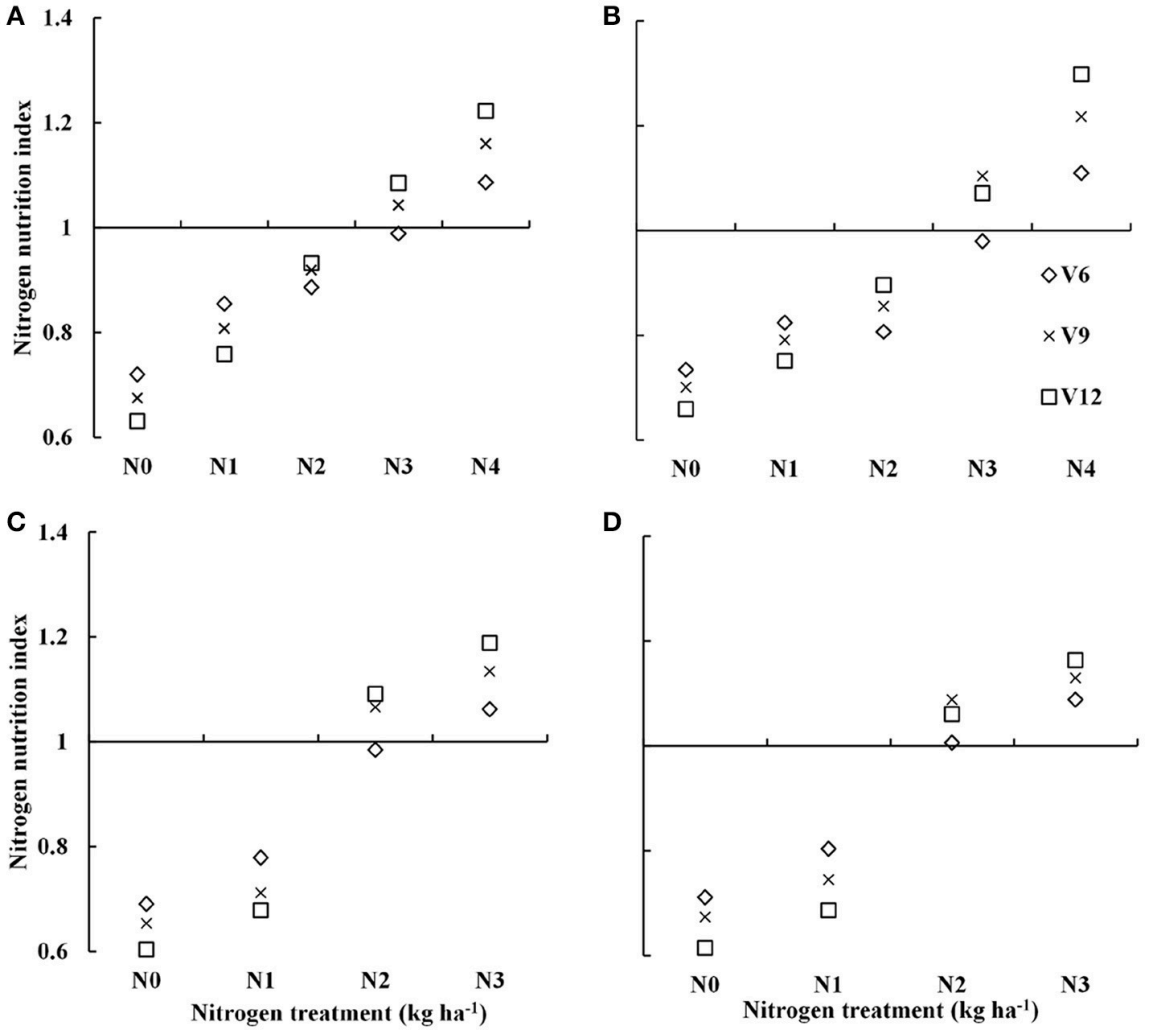
Dynamic changes in the nitrogen nutrition index (NNI) for summer maize under various nitrogen (N) application rates from V6 to V12 growth stages in experiments 1–4 [**(A)** 2015 DH605; **(B)** 2015 ZD958; **(C)** 2016 DH605; **(D)** 2016 ZD958]. V6, V9, and V12 represent the sixth ninth and twelfth leaf, respectively.

Nitrogen nutrition index showed substantial differences across the different N treatments (Figure 2). The NNI increased from V6 to V9 and to the V12 stage of summer maize with increasing N supply. The NNI under varied N rate treatments (0–300 kg N ha^−1^) for data acquired from experiment 1 to 4 were ranged from 0.60–1.22 and 0.61–1.3 for DH605 (Figures 2A,C) and ZD958 (Figures 2B,D) during 2015 and 2016 growing years, respectively. The NNI values of ~1 for N3 and N2 treatments (Table 1) during 2015 and 2016 growing years indicated an optimal N supply (Figure 2). The NNI values <1 for N0, N1, and N2 treatments and N0 and N1 treatments (Table 1) during 2015 and 2016 years, respectively indicated sub-optimal N growth conditions. In contrast, NNI values >1 for the N4 and N3 treatments (Table 1) during 2015 and 2016 year, respectively indicated supra-optimal N growth conditions. Additionally, the NNI values of N4 treatments tended to increase gradually from V6 to V12 stages, whereas the NNI values of N0 to N2 treatments tended to decrease gradually from V6 to V12 stages.

### Dynamic changes in leaf N concentration and leaf N gradient across different leaf layers with year, cultivar, growth stage, and N supply

The leaf N concentration varied from LL1 to LL3 for different cultivar, growth stages, and N application rates (Table 3). Leaf N concentration showed significant variations across the cultivars (ZD958 and DH605) and LLs (LL1 to LL3). The leaf N concentration of DH605 for each LL was significantly higher than that of ZD958. There was no significant difference between leaf N concentration of 2015 and 2016 years. The leaf N concentration of each LL gradually increased from N0 to N4 treatment. The lowest leaf N concentration of each LL was observed for N0 treatment while it was the highest for N4 treatments. The leaf N concentration of each LL gradually decreased toward advancing maturity of summer maize.

**Table 3:** Leaf nitrogen (N) concentration and leaf N gradient of two summer maize cultivars at four growth stages under five N levels during 2015 and 2016 growth years (experiment 1–4).

**Treatment**	**Leaf N concentration**			**Leaf N gradient**		
	**LL1**	**LL2**	**LL3**	**LL1–2**	**LL1–3**	**LL2–3**
**CULTIVAR (C)**						
ZD958	3.24b	3.05b	2.86b	0.19a	0.38a	0.19a
DH605	3.49a	3.3a	3.11a	0.19a	0.38a	0.19a
**YEAR (Y)**						
2015	3.41a	3.17a	2.94a	0.24a	0.47a	0.23a
2016	3.32a	3.18a	3.04a	0.14b	0.28b	0.14b
**N TREATMENT (N)**						
N0	2.94d	2.71d	2.47d	0.23a	0.47a	0.24a
N1	3.23c	3.03c	2.84c	0.2b	0.39b	0.19b
N2	3.48b	3.29b	3.12b	0.19c	0.36c	0.17c
N3	3.59ab	3.42ab	3.26ab	0.17cd	0.33c	0.16c
N4	3.84a	3.68a	3.5a	0.16d	0.34c	0.18c
**GROWTH PERIOD (G)**						
V6	3.6a	3.4a	3.2a	0.2a	0.4a	0.2a
V9	3.37a	3.17ab	2.98ab	0.2a	0.39a	0.19a
V12	3.14b	2.96b	2.77b	0.18a	0.37a	0.19a
C × Y	^*^	^*^	^*^	NS	NS	NS
C × N	^*^	^*^	^*^	^*^	^*^	^*^
C × G	^*^	^*^	^*^	NS	NS	NS
Y × N	^*^	^*^	^*^	^*^	^*^	^*^
Y × G	^*^	^*^	^*^	NS	NS	^*^
N × G	NS	NS	NS	NS	NS	NS
C × Y × N	^*^	^*^	^*^	NS	NS	NS
C × Y × G	NS	NS	NS	^*^	NS	NS
Y × N × G	NS	NS	NS	NS	NS	NS

LL, leaf layer. LL1, LL2, and LL3 indicate the leaf N concentration of different LLs of the canopy, respectively. LL1-2, LL1-3, and LL2-3 indicate the difference in leaf N concentration between different LLs of the canopy, respectively. V6, V9, and V12 indicate the sixth, ninth and twelfth fully expanded leaf of more than 50% plant in the field, respectively. Different letters between columns in the same row indicate a significant difference (p < 0.05). NS refers to no significant differences between treatments at 0.05 level.

* *Refers to significant differences between treatments at 0.05 level*.

The leaf N gradient varied across the year and N application rates (Table 3). There was no significant difference observed for the leaf N gradient of each LL between cultivars (ZD958 and DH605). The leaf N gradient of each LL during 2015 was significantly higher year than that in 2016. A significant decline in leaf N gradient was observed with increasing N supply at the same LL. The highest leaf N gradient (0.47) was observed for LL1–3 under N0 treatment. In contrast, the lowest leaf N gradient (0.16) for LL1–2 and LL2–3 were observed under N4 and N3 treatments, respectively. The leaf N gradient for each LL showed non-significant differences for different crop growth stages (V6 to V12).

### Dynamic changes in the CM readings, CM gradients, and PDCMI among different leaf layers with years, cultivars, growth stages, and N supply

The CM readings showed significant variation across the growing years and N application rates (Table 4). However, there was no significant difference between the CM readings of DH605 and ZD958. The variation in the CM readings was obvious during 2015 as compared to those of 2016. Moreover, no significant variation was observed for CM readings at different growth stages. The CM readings of different LLs increased with increasing N supply under the N-deficient condition, yet this increase in CM readings was minor under N-sufficient condition. The vertical decline in CM readings of different LLs (CM1 to CM3) showed the similar trends that were for leaf N concentration of different LLs. The difference between CM1 and CM3 was 10.4 and 5.7 for N0 and N4 treatments, respectively.

**Table 4:** Chlorophyll meter (CM) readings, CM gradient and positional difference chlorophyll meter index (PDCMI) of two summer maize cultivars at the three growth stages under five nitrogen (N) levels during 2015 and 2016 growing years (experiment 1–4).

**Treatment**	**CM reading**			**CM gradient**			**PDCMI**		
	**CM1**	**CM2**	**CM3**	**CM1-2**	**CM1-3**	**CM2-3**	**PDCMI1-2**	**PDCMI1-3**	**PDCMI2-3**
**CULTIVAR (C)**									
ZD958	45.2a	41.5a	36.8a	3.7a	8.4a	4.6a	0.04a	0.1a	0.06a
DH605	46.8a	43.2a	38.1a	3.6a	8.7a	5a	0.04a	0.1a	0.06a
**YEAR (Y)**									
2015	47.3a	43.4a	39a	3.8a	8.3a	4.5b	0.04a	0.1a	0.06a
2016	44.3b	40.9b	35.7b	3.4a	8.6a	5.2a	0.04a	0.11a	0.06a
**N TREATMENT (N)**									
N0	41.7d	37.5c	31.3d	4.2a	10.4a	6.2a	0.05a	0.14a	0.09a
N1	44.2c	40.1b	35c	4.1a	9.2a	5.1b	0.05a	0.12b	0.07b
N2	47.1b	45.8a	38.6b	3.9ab	8.6b	4.5c	0.04ab	0.1c	0.06c
N3	48.9a	45.8a	41.7a	3.1b	7.2b	4.1c	0.03b	0.08d	0.05cd
N4	49.9a	47.6a	44.2a	2.3c	5.7c	3.4d	0.02c	0.06e	0.04d
**GROWTH PERIOD (G)**									
V6	45a	41.2a	36.6a	3.8a	8.4a	4.6a	0.04a	0.1a	0.06a
V9	45.7a	41.8a	37a	3.9a	8.7a	4.8a	0.05a	0.11a	0.06a
V12	47.2a	43.9a	39a	3.3a	8.2b	4.9a	0.04a	0.1a	0.06a
C × Y	^*^	^*^	^*^	NS	NS	^*^	NS	^*^	^*^
C × N	^*^	^*^	^*^	NS	^*^	NS	NS	NS	NS
C × G	^*^	^*^	^*^	NS	NS	NS	NS	^*^	NS
Y × N	NS	NS	NS	NS	^*^	NS	NS	^*^	NS
Y × G	NS	NS	NS	NS	NS	^*^	NS	^*^	^*^
N × G	NS	NS	NS	NS	NS	NS	NS	NS	NS
C × Y × N	NS	NS	NS	NS	NS	NS	NS	NS	NS
C × Y × G	NS	NS	NS	NS	NS	NS	NS	NS	NS
Y × N × G	NS	NS	NS	NS	NS	NS	NS	NS	NS

CM1, CM2, and CM3 indicate the CM readings of LL1, LL2 and LL3, respectively. CM1-2, CM1-3, and CM2-3 indicate the difference in CM is reading between different LLs of the canopy, respectively. PDCMI1-2, PDCMI1-3, and PDCMI2-3 indicate the difference in PDCMI between different LLs of the canopy, respectively. V6, V9, and V12 indicate the sixth, ninth and twelfth fully expanded leaf of more than 50% plant in the field, respectively. Different letters between columns in the same row are significantly different (p < 0.05). NS refers to no significant differences between treatments at 0.05 level.

* *Refers to significant differences between treatments at 0.05 level*.

The significant difference of CM gradient was observed for different N treatments (Table 4). The highest values (4.2, 10.4 and 6.2) and the lowest values (2.3, 5.7, and 3.4) of CM1–2, CM1–3, and CM2–3 were observed under N0 and N4 treatments, respectively. CM gradient decreased gradually from low to high N treatments. There was a significant difference for CM1–3 from V6 to V12 growth stage, but CM1–2 and CM2–3 values had non-significant differences for the same growth period.

The ranges and changes in PDCMI values under varied N rate treatments are shown in Table 4. PDCMI values of different LLs showed non-significant differences between cultivars, years, and growth stages. The PDCMI1–2, PDCMI1–3, and PDCMI2–3 values decreased gradually from N0 to N4 treatments. The highest (0.14) and the lowest (0.02) PDCMI values were observed under the N0 treatment for PDCMI1–3 and for PDCMI1–2 under N4 treatment, respectively. The PDCMI1–3 values were higher than the other two indices for the same N treatment (Table 4).

### The relationships between leaf N gradient and CM gradient, NNI across different leaf layers

**Figure 3:**
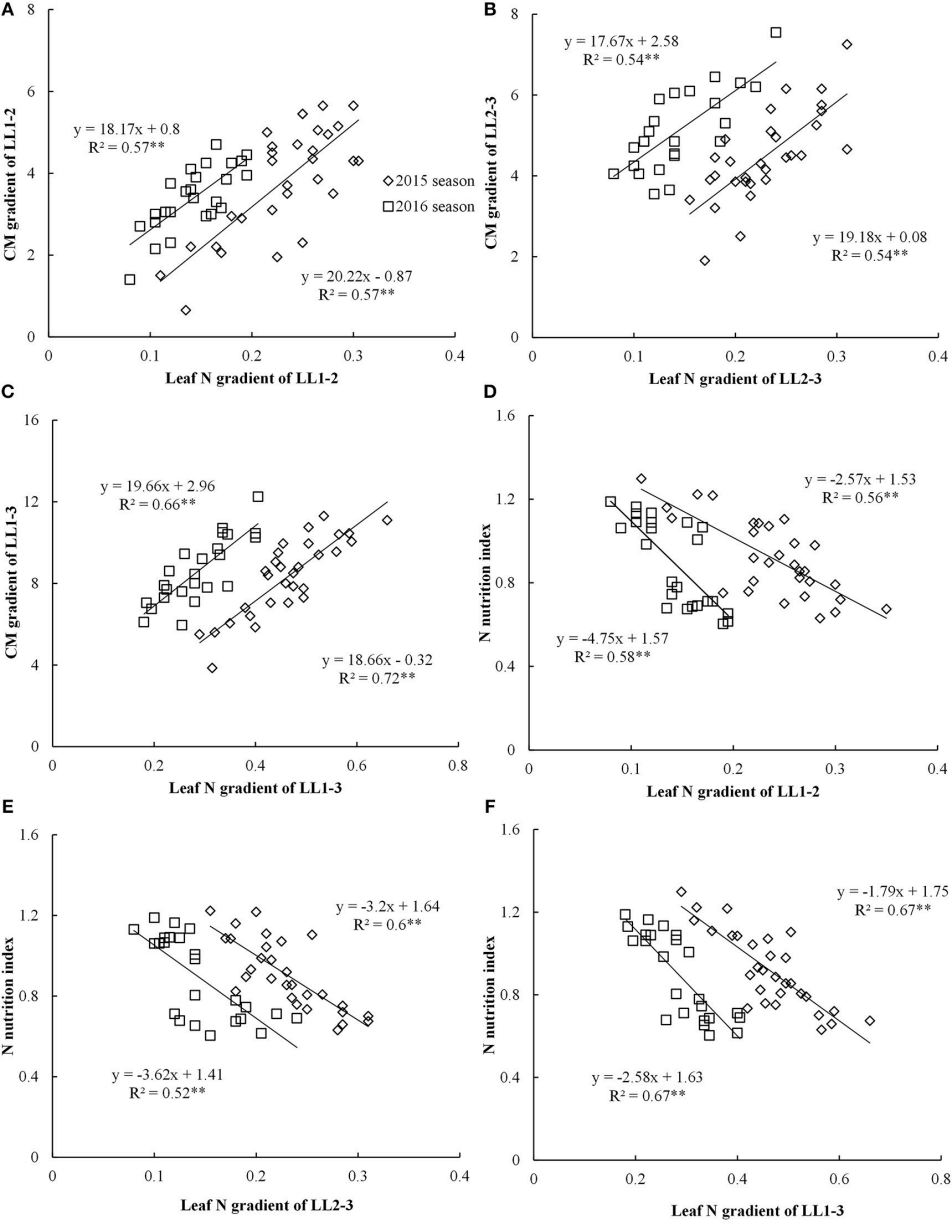
Relationships between leaf nitrogen (N) gradient and chlorophyll meter (CM) gradient, nitrogen nutrition index (NNI) in different leaf layers (LLs) from V6 to V12 growth stages during the 2015 and 2016 years [**(A)** LL1-2 vs. CM1-2; **(B)** LL2-3 vs. CM2-3; **(C)** LL1-3 vs.CM1-3; **(D)** LL1-2 vs. NNI; **(E)** LL2-3 vs. NNI; **(F)** LL1-3 vs. NNI]. V6 and V12 represent the sixth and twelfth leaf, respectively. LL1–2, LL1–3, and LL2–3 indicate the difference in leaf N concentration between different LLs of the canopy, respectively. CM1-2, CM1-3, and CM2-3 indicate the difference in CM is reading between different LLs of the canopy, respectively.

The CM gradient of each LL (LL1 to LL3) showed significant positive linear relationships with leaf N gradient of the corresponding LL, yet these relationships were influenced by growing years (Figures 3A–C). The coefficient of determination (*R*^2^) values for these relationships were ranged from 0.54 to 0.72 across different LLs. The relationships between leaf N gradient and CM gradient of LL1–3 with *R*^2^-value ranging from 0.66 to 0.72 were stronger than the relationships of LL1–2 and LL2–3.

The significant negative linear relationships between NNI and leaf N gradient of the different LLs (LL1 to LL3) were noticed (Figure 3). However, these relationships were influenced by growing year (Figures 3D–F). The *R*^2^-values ranged from 0.56 to 0.67 across different LLs. The performance (*R*^2^-value of 0.67) of the relationships between leaf N gradient of LL1–3 and NNI was better than those of other two relationships of LL1–2 and LL2–3.

### Development and validation of the relationships between CM readings and NNI across different leaf layers

**Figure 4:**
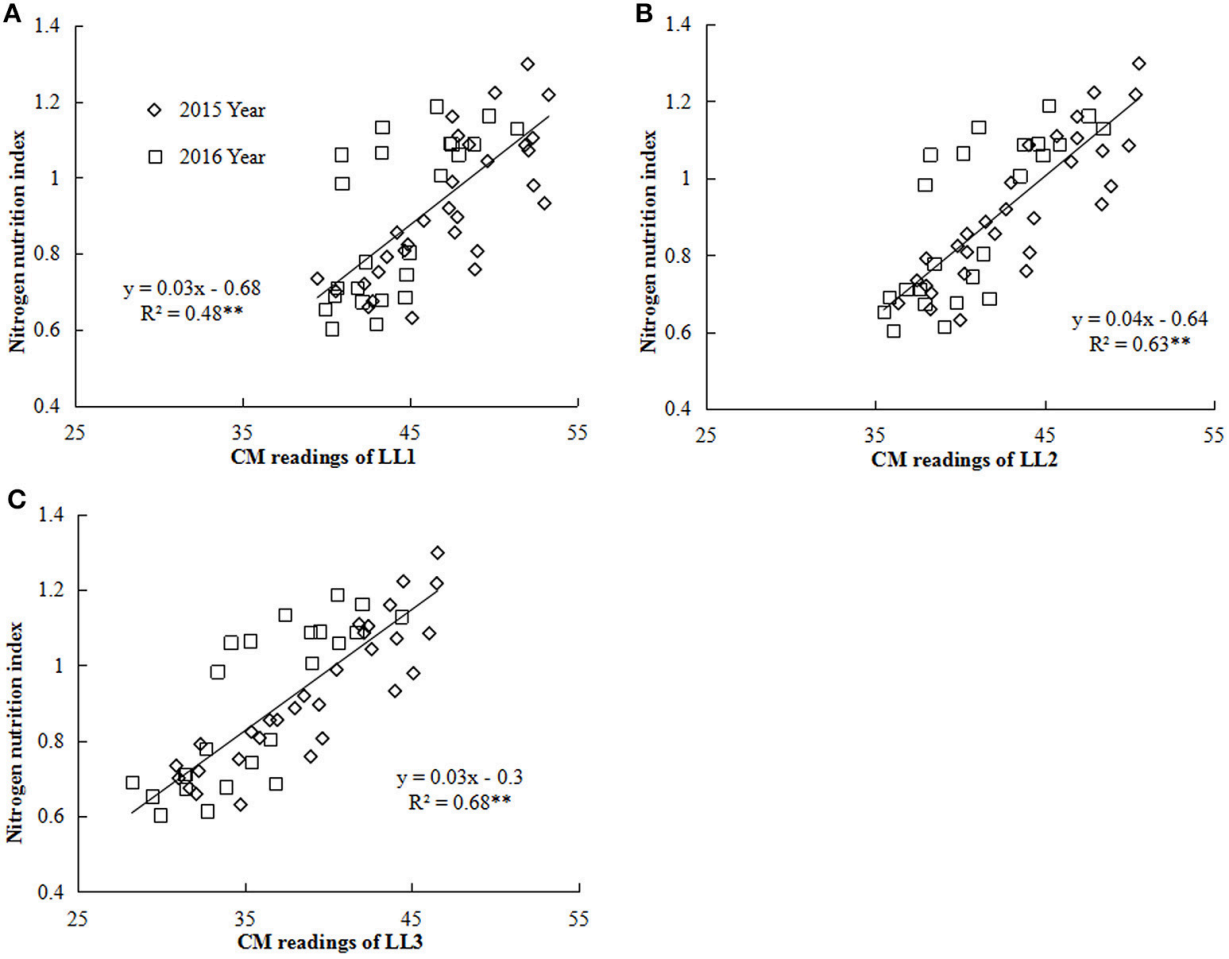
Relationship between chlorophyll meter (CM) readings of different leaf layers (LLs) and NNI (nitrogen nutrition index) from V6 to V12 growth stages of summer maize during the 2015 and 2016 years [**(A)** LL1 vs. NNI; **(B)** LL2 vs. NNI; **(C)** LL3 vs. NNI]. V6 and V12 represent the sixth and twelfth leaf, respectively. LL1, LL2, and LL3 indicate the leaf N concentration of different LLs of the canopy, respectively.

Nitrogen nutrition index and he CM readings of each LL (from LL1 to LL3) showed significantly positive linear relationships (Figure 4). The *R*^2^-values for these relationships were ranged from 0.48 to 0.68 and the strongest relationship with the *R*^2^-value of 0.68 were observed for the LL3 (Figure 4). In the present study, a simple linear regression with the groups was used to determine the differences in the slope and intercept of the linear regression models between the NNI and CM readings (LL1 to LL3) across the years and cultivars (Table 5). The results showed that the intercepts (*b*) of the linear regression models between NNI and CM readings for LL1, LL2, and LL3 were significantly different in two growing years (*P* < 0.05). Therefore, the linear regression models between NNI and CM readings of the different LLs (LL1 to LL3) were easily affected by year.

**Table 5:** Simple linear regression with groups between the chlorophyll meter (CM) readings of different leaf layers (LLs) and the nitrogen nutrition index (NNI) by using the data acquired from experiments 1 to 4.

**Parameter**	**Factor**	***t*** **(50) value**		
		**CM1**	**CM2**	**CM3**
Slope (*a*)	Year	0.56^ns^	0.53^ns^	0.86^ns^
	Cultivar	−1.31^ns^	−1.82^ns^	1^ns^
Intercept (*b*)	Year	2.21^*^	2.21^*^	2.94^**^
	Cultivar	0.55^ns^	1.27^ns^	−1.16^ns^
*P* (*0.05*) value			2.009	
*P* (*0.01*) value			2.678	

CM1, CM2, and CM3 indicate the CM readings of LL1, LL2 and LL3, respectively.

* Indicates significant at the 0.05 level.

** *Indicates significant at the 0.01 level. ns indicates no significant*.

### Development and validation of the relationship between the PDCMI and NNI across different leaf layers

**Figure 5:**
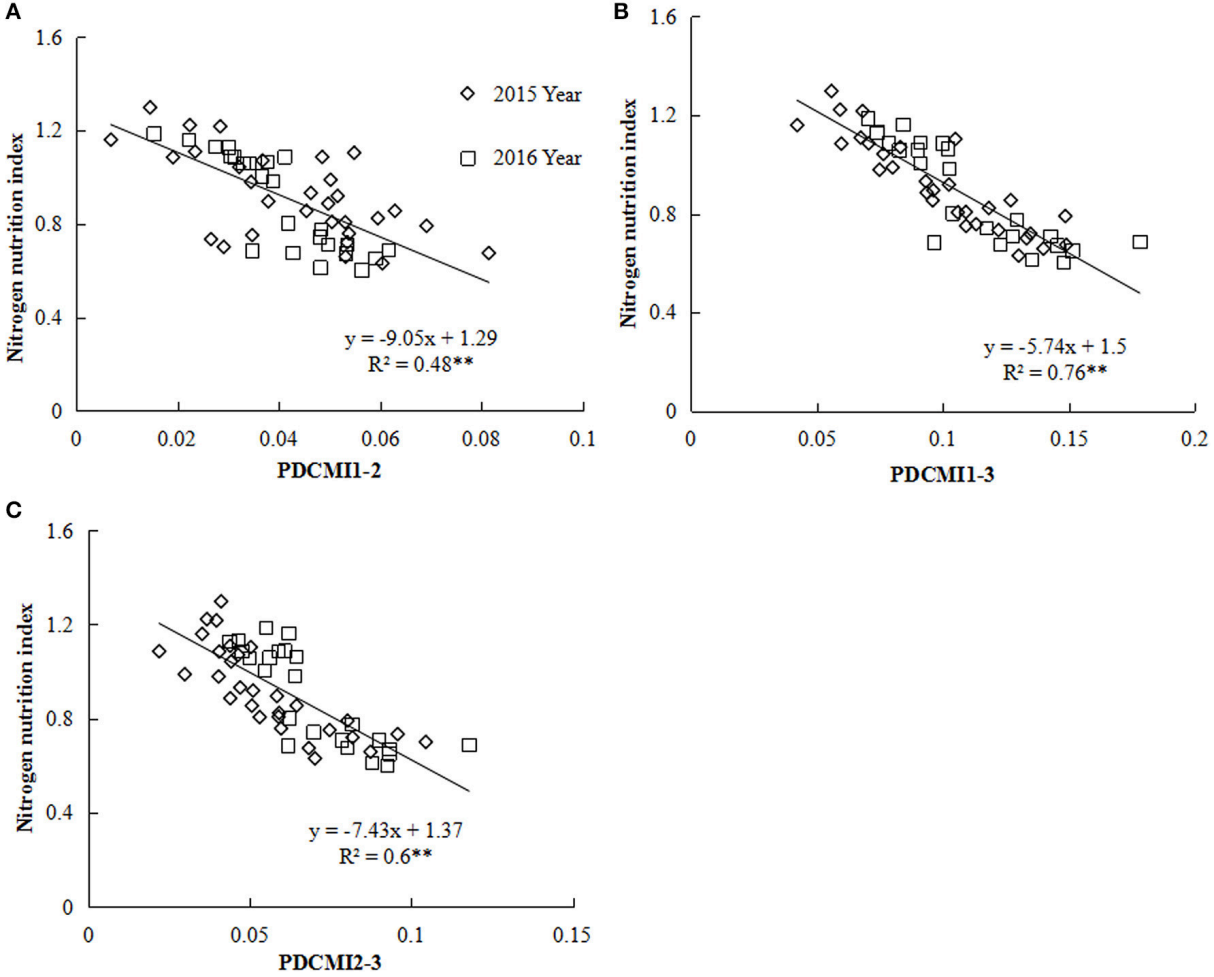
Relationship between the positional difference chlorophyll meter index (PDCMI) of different leaf layers (LLs) and the nitrogen nutrition index (NNI) from V6 to V12 growth stages of summer maize during 2015 and 2016 years [**(A)** PDCMI1-2 vs. NNI; **(B)** PDCMI1-3 vs. NNI; **(C)** PDCMI2-3 vs. NNI]. V6 and V12 represent the sixth and twelfth leaf, respectively. PDCMI1-2, PDCMI1-3, and PDCMI2-3 indicate the normalized value between CM readings of different LLs, respectively.

**Figure 6:**
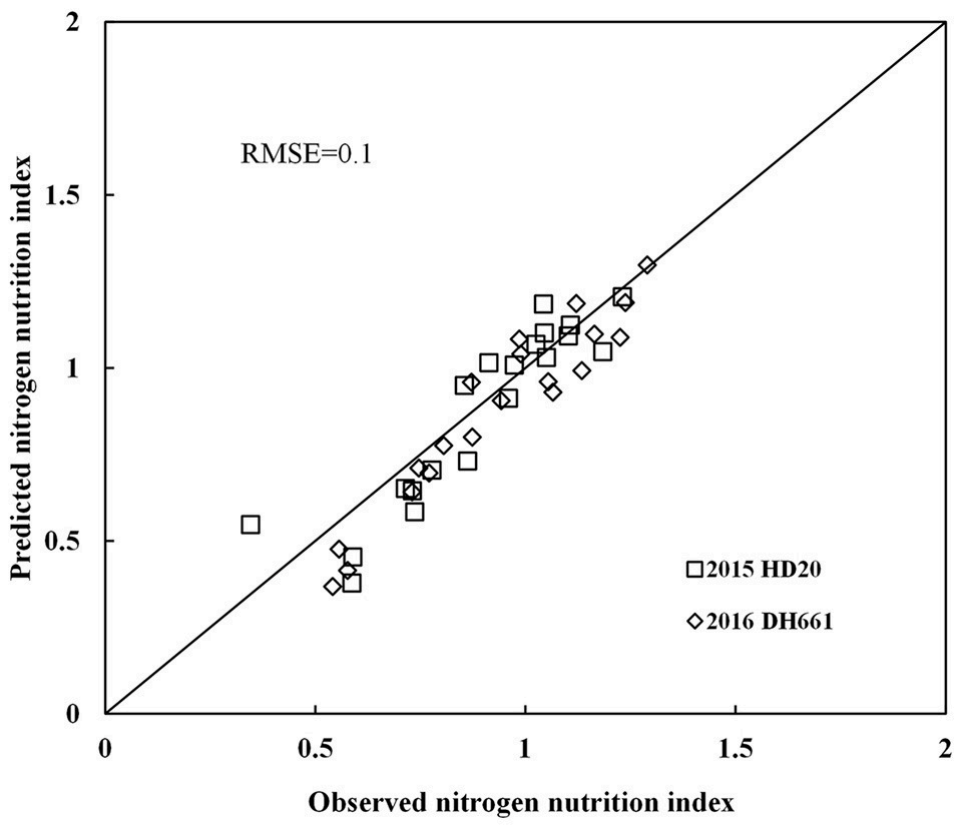
Comparison of observed and predicted nitrogen nutrition index (NNI) by using independent data acquired from experiments 5 to 6.

Nitrogen nutrition index and PDCMI of different LLs showed significantly negative linear relationships (Figure 5). The *R*^2^-values for these relationships were ranged from 0.48 to 0.76. The strongest relationship with *R*^2^-values of 0.76 was observed for NNI-PDCMI1–3 relationship. The lower *R*^2^-values between NNI-PDCMI1–2 and NNI-PDCMI2–3 relationships indicated that these relationships could not distinguish the change in the NNI across the N treatments. In this study, a simple linear regression with groups was used to determine the differences in the slope and intercept of the linear regression models between the NNI and PDCMI of different LLs (LL1 to LL3) across the years and cultivars (Table 6). The results indicated that the slopes (*a*) of the PDCMI1–2 and the intercept (*b*) of PDCMI2–3 were significantly different between two growing years (*P* < 0.05). There was no significant difference in the slope (*a*) and intercept (*b*) of PDCMI1–3 across the years and cultivars. The PDCMI1–3 between the upper and lower LLs effectively eliminated the effects of environmental factors on the CM readings, which in turn can improve the estimation accuracy of NNI (Figure 5). The data of two independent experiments (5 and 6) were used to validate the prediction accuracy of the linear regression model between PDCMI1–3 and NNI (*y* = −5.74x + 1.5, *R*^2^ = 0.76^**^). The results showed that RMSE value between the predicted and observed NNI values were 0.1 for PDCMI1–3 (Figure 6).

**Table 6:** Simple linear regression with groups between positional difference chlorophyll meter index (PDCMI) of different leaf layers and the nitrogen nutrition index (NNI) by using the data acquired from experiments 1 to 4.

**Parameter**	**Factor**	***t*** **(50) value**		
		**PDCMI1–2**	**PDCMI1–3**	**PDCMI2–3**
Slope (*a*)	Year	−3.01^**^	−0.5^ns^	−0.93^ns^
	Cultivar	0.74^ns^	−1.1^ns^	−1.08^ns^
Intercept (*b*)	Year	−1.27^ns^	1.42^ns^	2.41^*^
	Cultivar	−0.55^ns^	0.3^ns^	0.91^ns^
*P* (*0.05*) value			2.009	
*P* (*0.01*) value			2.678	

PDCMI1-2, PDCMI1-3, and PDCMI2-3 indicate the normalized value between CM readings of different LLs, respectively.

* Indicates significant at the 0.05 level.

** *Indicates significant at the 0.01 level. ns indicates no significant*.

## Discussion

The results of the present study indicated that NNI has well reflected the plant N status from V6 to V12 growth stages of summer maize The NNI values <1, around 1 and >1 under sub-optimal, optimal and supra-optimal N nutrition in present study (Figure 2) were in consensus with previous reports on rice, wheat, and maize grown in different regions (Yao et al., 2014b; Ata-Ul-Karim et al., 2017c; Zhao et al., 2017). For the practical application of NNI for crop N status diagnosis, CM readings were used to estimate NNI values summer maize. Previous studies have confirmed that the field CM readings had a significant positive relationship with leaf N concentration of a given maize cultivar and location (Wood et al., 1992). However, due to the unique greenness characteristics of different maize cultivars, the calibration of absolute CM readings might not be practical to determine crop N status (Schepers et al., 1992). The results of present study showed that CM readings of each LL (LL1, LL2, and LL3) well-reflected leaf N concentration of the corresponding LL under different N treatments, but CM readings were influenced by years (Table 4). These results were in consensus with a previous report on wheat (Prost and Jeuffroy, 2007). Additionally, the non-significant difference of CM readings under high N treatments in maize (Table 4) was in agreement with that reported on maize by Ziadi et al. (2008). The non-significant difference of CM readings under high N treatments refers to the fact that the maize leaf usually contain 2–3% of the N as nitrate and that most of the extra N is stored in the stem as nitrate and the increasing N in the nitrate form was not detected by CM at the high N treatments (Prost and Jeuffroy, 2007). Therefore, the relationships between NNI and CM readings of each LL were relatively weaker (Table 5) and were unstable across the years (2015 and 2016). The relatively weaker relationships between CM and NNI were also reported in the different crops (Ziadi et al., 2008; Yang et al., 2014; Zhao et al., 2016). Therefore, the CM readings of single leaf position or LL are not suitable to estimate NNI across different external environments (Zhao et al., 2016).

The vertical decline in leaf N concentration from LL1 to LL3 with the canopy development of summer maize in the present study was well justified by the crop N dilution and canopy photosynthesis optimization theories (Hirose and Werger, 1987; Lemaire et al., 2008). During the crop growth, the increasing proportion of structural tissues (stem) allows the summer maize plant to grow taller for capturing more light radiation. Due to the higher light intensity in the top leaves, as compared to the bottom leaves, plant N was transferred from bottom to top leaves which in turn maximizes the photosynthetic capacity of crop canopy (Li et al., 2013). Therefore, a non-uniform N distribution existed between the top and bottom leaves of the canopy. Moreover, the decline in leaf N concentration from V6 to V12 stages (Table 3) was also related to the non-uniform N distribution in the canopy and was in consensus with the previous report on rice or wheat (Yao et al., 2014a). The increased proportion of bottom leaves with low leaf N concentration and the decreased proportion of top leaves with high leaf N concentration toward advancing maturity of summer maize, was in consensus with previous reports (Yao et al., 2014a,b; Zhao et al., 2017). This phenomenon resulted in the declined leaf N concentration of the whole canopy during the growth period of summer maize (V6 to V12). The decline in (0.47 and 0.34) leaf N gradient from LL1 to LL3 under the N0 and N4 treatments indicated that the decline of leaf N gradient between the top and bottom LLs is faster in the N0 treatment as compared to the N4 treatment. The previous study reported that maize has a more uniform distribution of light and leaf N throughout the canopy (Lemaire et al., 2008). Leaf N gradient of summer maize canopy in this study was more uniform under adequate N supply due to the better light environment of the bottom LL. The transfer and storage of absorbed N in the bottom LL can minimize the leaf N gradient between the top and bottom LL under the ample N supply (Yuan et al., 2016). However, under sub-optimal N condition, summer maize plant could not absorb enough N from the soil during the growth period and N from bottom LL was transported to the top LL to fulfill the demand for canopy photosynthesis according to the crop N dilution and canopy photosynthesis optimization theories (Hirose and Werger, 1987; Lemaire et al., 2008). The leaf N gradient within the summer maize canopy gets steeper due to the decline in plant N (Table 3, Dreccer et al., 2000). Therefore, the bottom LL may reflect a better plant N status than the top LL in summer maize across varied N application rates. Another report on rice also showed that the increase in leaf N gradient of the bottom leaves was higher than that of the top leaves under increasing varied N supply (Wang et al., 2006).

The PDCMI in the present study has effectively eliminated the effects of year, cultivar, and growth stage on CM readings. The results of the gradual decline in PDCMI with the increasing N application rates were in consensus with the previous report on barley and indicated that N rate could reduce the difference between CM readings of different LLs (Zhao et al., 2016). The significantly negative linear regression model between NNI and PDCMI13 was stable across different cultivars, sites and years. However, Yu et al. (2012) reported the ratio of CM readings of different leaves (the CM readings ratio between the first and third fully expanded leaves from the top) to diagnose maize N status, and their results showed that the ratio of CM reading of different leaves is not a suitable method to diagnose summer maize N status. This is because the first and third leaves are on the upper canopy of maize, which is less sensitive than the bottom leaves to the change of maize N status. In this study, PDCMI1–3 integrated the CM readings information within the upper and bottom leaves of the summer maize canopy. LL1 was not influenced by leaf aging and the progressive shading by newer leaves because it was located on the top of the canopy (Ziadi et al., 2008). Therefore, CM readings of LL1 might be not easily affected by summer maize growth (Gastal et al., 2001). The CM readings of LL3 were more sensitive to the change in plant N status across various N rates in summer maize (Table 4). The determination of PDCMI13 is rapid and simpler than the existing methods of using CM readings as a diagnostic tool because its measurement did not require a fully N fertilized treatment as a control to estimate NNI. Although the models developed in this study were validated with the data acquired from independent experiments (Figure 6), yet further investigation is required to test the applicability and reliability of the present model between PDCMI1–3 and NNI under different climatic conditions. Further investigations would be helpful for developing an efficient, rapid, and non-destructive approach to accurately assess the crop N status for precisely managing N application during the growth period of summer maize crop.

## Conclusions

Leaf N concentration of LL3 can better reflect the plant N status of summer maize than those of the other two LLs. The leaf N gradient of each LL and NNI showed significantly positive linear relationships with the CM readings gradient of the corresponding LL while it showed a significantly negative linear relationship with NNI. The strongest relationship was observed between NNI and CM readings of the LL3 with the *R*^2^-value of 0.68. Nevertheless, the linear relationship was affected by the growing year. The significantly negative linear relationships between NNI and PDCMI of the different LLs were observed and the relationship between PDCMI1–3 and the NNI was stable across the cultivars, sites, and growing years. The relationship between PDCMI1–3 and NNI (*y* = −5.74x + 1.5, *R*^2^ = 0.76) was the most accurate and stable across the growing year and cultivars, hence can be implemented for a rapid and simple diagnosis of plant N status of summer maize cultivation in China.

## Author contributions

BZ and AD: conceived the idea and led the study design; BZ, SA-U-K, and ZhL: carried out the experiment, performed analysis and wrote the paper; JZ, JX, ZuL, and AQ: assisted with study design and experiments; DN, QY, and YZ: edited the manuscript.

### Conflict of interest statement

The authors declare that the research was conducted in the absence of any commercial or financial relationships that could be construed as a potential conflict of interest.
